# A multifunctional enzyme portfolio for α-chaconine and α-solanine degradation in the *Phthorimaea operculella* gut bacterium *Glutamicibacter halophytocola* S2 encoded in a trisaccharide utilization locus

**DOI:** 10.3389/fmicb.2022.1023698

**Published:** 2022-10-12

**Authors:** Wenqian Wang, Guangzu Du, Guangyuan Yang, Ke Zhang, Bin Chen, Guanli Xiao

**Affiliations:** ^1^State Key Laboratory for Conservation and Utilization of Bio-Resources in Yunnan, College of Plant Protection, Yunnan Agricultural University, Kunming, China; ^2^College of Agronomy and Biotechnology, Yunnan Agricultural University, Kunming, China

**Keywords:** *Glutamicibacter halophytocola*, glycoalkaloids degradation, α-chaconine, α-solanine, α-rhamnosidase, β-galactosidase, β-glucosidase

## Abstract

Steroidal glycoalkaloids (SGAs) are secondary metabolites commonly found in members of the family Solanaceae, including potatoes, and are toxic to pests and humans. The predominant SGAs in potato are α-chaconine and α-solanine. We previously reported that *Glutamicibacter halophytocola* S2, a gut bacterium of the pest *Phthorimaea operculella* (potato tuber moth), can degrade α-chaconine and α-solanine in potatoes, which can improve the fitness of *P. operculella* to feed on potatoes with a high content of toxic SGAs. *Glutamicibacter halophytocola* S2 harbored a gene cluster containing three deglycosylase genes—*GE000599*, *GE000600*, and *GE000601—*that were predicted encode α-rhamnosidase (RhaA), β-glucosidase (GluA), and β-galactosidase (GalA). However, there is limited information is available on the enzyme activities of the three enzymes expressed by this gene cluster and how they degrade the major toxic α-chaconine and α-solanine. In the current study, each enzyme of this gene cluster was produced by a prokaryotic expression approach and the activity of the recombinant enzymes for their target substrate and α-chaconine and α-solanine were evaluated by EPOCH microplate spectrophotometer and liquid chromatography mass spectrometry (LC-MS). The three enzymes had multifunctional activities, with RhaA and GluA could hydrolyze α-rhamnose, β-glucose, and β-galactose, while GalA can hydrolyze β-glucose and β-galactose. The degradation of α-chaconine and α-solanine was consistent with the results of the enzyme activity assays. The final product solanidine could be generated by adding RhaA or GluA alone. In conclusion, this study characterized the multifunctional activity and specific degradation pathway of these three enzymes in *G. halophytocola* S2. The three multifunctional enzymes have high glycosidic hydrolysis activity and clear gene sequence information, which help facilitates understanding the detoxification mechanism of insect gut microbes. The enzymes have a broad application potential and may be valuable in the removal of toxic SGAs from for potato food consumption.

## Introduction

Steroidal glycoalkaloids (SGAs) are natural nitrogen-containing specialized secondary metabolites and commonly exist in the plants of the family Solanaceae (e.g., potato, tomato, eggplant, and so on) (Friedman, 2006; Ryota et al., 2022). α-Solanine and α-chaconine are two major SGAs that account for 95% of the total SGAs content in potato tubers (Ha et al., 2012). These two SGAs consist of a non-polar lipophilic steroid nucleus extended by two fused nitrogen-containing heterocyclic rings at one end and bound to a polar water-soluble carbohydrate component at the other. The side-chain of α-solanine is composed of the trisaccharide solatriose ((α-L-rhap(1-2)[β-D-Glcp(1-3)] β-D-galactopyranosyl) and that of α-chaconine is composed of chacotriose ((α-L-rhap(1-2)[α-L-rhapGlcp1-2)] β-D-glucopyranyl) (Friedman and McDonald, 1997). α-Solanine and α-chaconine are toxic to bacteria, fungi, viruses, insects, animals, and humans, and aids in plant protection (Tingey, 1984; Fewell and Roddick, 1993; Korpan et al., 2004; Yamashoji and Matsuda, 2013; Lin et al., 2018). Much ingestion of SGAs can cause to nausea, fever, diarrhea, headache, and hallucinations (Grunenfelder et al., 2006). The potential human toxicity of SGAs has led to the establishment of guidelines limiting the SGAs content of new cultivars of potatoes before they can be released for commercial use (Liu et al., 2020). Following harvest, the glycoalkaloid content can increases during storage and transportation and under the influence of light, heat, cutting, slicing, sprouting, and exposure to phytopathogens (Deng et al., 2020). At present, the main methods to reduce the content of solanine are applying sprout suppressant (chlorpropham, CIPC), genetic modification, and controlling storage conditions. However, there are obvious deficiencies in both chemical and genetic modification methods (Smith and Bucher, 2012; Sawai et al., 2014). Therefore, it is crucial to identify a safer strategy to reduce the content of solanine in potatoes.

α-Solanine and α-chaconine are reported to be degraded by acid hydrolysis or enzymatic hydrolysis (Friedman et al., 1993; Weltring et al., 1997). Chemical reactions such as acid hydrolysis and two-phase acid hydrolysis require a temperature as high as 85°C and generate a large amount of chemical waste. In addition, because of its low selectivity and yield, the chemical method to control SGAs in potatoes has been abandoned (Li et al., 2019). Some enzymes in potato-derived extracts and extracts of fungus-derived pathogens contain α-solanine and α-chaconine degradation activities (Weltring et al., 1997; Nikolic and Stankovic, 2005; Dahlin et al., 2017), which can remove the carbohydrate by stepwise degradation. And the complete deglycosylation of α-solanine and α-chaconine requires the participation of multiple glycoside hydrolases (GHs). Enzymes are considered natural products, and the discovery of unique functions of enzymes is very beneficial to the development of environmentally friendly and sustainable use of resources (Fernandes, 2010; Cheng et al., 2021). At present, limited information is available on the microbes involved in the degradation of α-solanine and α- chaconine and the use of microbial enzymes. Oda et al. (2002) found that *Plectosphaerella cucumerina* could only produce α-L-rhamnosidase, which could degrade α-chaconine to β_1_-chaconine, but could not degrade α-solanine. Jensen et al. (2009) reported that microorganisms in groundwater could stepwise hydrolyze α-solanine into β-solanine, γ-solanine and solanidine, and degrade α-chaconine into β-chaconine, γ-chaconine, and solanidine. The toxicity of these products decreased sequentially, but the microbial species involved in the process were not identified. Hennessy et al. (2018) found that *Arthrobacter* and *Serratia* could efficiently degrade the glycosidic alkaloids α-chaconine and α-solanine by three deglycosylation enzymes β-galactosidase (GalA), β-glucosidase (GluA), and α-rhamnosidase (RhaA) belonging to the GH2, GH3, and GH78 protein families, respectively. The enzymatic activity of the gene cluster involved in the complete deglycosylation of both α-chaconine and α-solanine was identified (Hennessy et al., 2020). However, the effects of each enzyme alone or jointly were not evaluated to determine the different steps involved in the degradation of both compounds.

We recently isolated and described *Glutamicibacter halophytocola* S2, a bacterium capable of complete degradation of SGAs in the *Phthorimaea operculella* gut, which can improve the fitness of *P. operculella* to feed on potatoes with a high content of toxic SGAs. Moreover, a gene cluster comprising three genes encoding RhaA, GluA, and GalA was identified in *G. halophytocola* S2, and these genes had higher expression on α-solanine medium (Wang et al., 2022). The present study aimed to evaluate the role of the enzymes encoded by these three genes, both as each enzyme alone or jointly to determine the different steps involved in the degradation of α-chaconine and α-solanine. The three enzymes described in this paper have potential application value in the removal of toxic glycosidic alkaloids and help us to enhance understanding of the detoxification mechanism of insect gut microorganisms.

## Materials and methods

### Strains and culture conditions

Bacterial strains were routinely cultured in liquid or solid (1.5% agar) lysogeny broth (LB) medium at 25°C (*G. halophytocola* S2) or 37°C *(Escherichia coli* for cloning and expression). When required, the medium was supplemented with 100 μg/mL ampicillin.

### Gene cloning and production of recombinant proteins

Genomic DNA of *G. halophytocola* S2 was extracted using the freeze-thaw method according to Zheng et al. (2017). Primers were designed to target complete gene sequence without any predicted signal peptides (Table 1). Three genes were amplified from *G. halophytocola* S2 genomic DNA by polymerase chain reaction (PCR) using Q5 High-Fidelity DNA polymerase (NEB, USA) and the respective forward and reverse primers. PCR products were purified using the E.Z.N.A. Cycle-Pure Kit (Omega, USA). Plasmid pET15b (Novagen, USA) harboring the ampicillin selectable marker and an N-terminal HIS tag was modified and subsequently used as a cloning and heterologous expression vector (Hennessy et al., 2020). Plasmid extraction was performed using Plasmid Mini kit I (Omega). Three genes construct was cloned into the *Nde*I site of a pET15b vector (Novagen). The digestion products were recycled by Gel Extraction Kit (Omega). A One Step Cloning Kit (Vazyme, China) was used for recombination.

**TABLE 1:** Primers used for amplification of the target genes from the genomic DNA of *Glutamicibacter halophytocola* S2.

Gene name	Primer sequence (5′–3′)	Nucleotides
*GE000599*	F: gtgccgcgcggcagccatatgGTGATGACCGAGTCCAGTTCT	1–2,487
	R: ggctttgttagcagccggatcTCACTTGTTGGCTCCTTCAAG	
*GE000600*	F: gtgccgcgcggcagccatatgGTGAACACCTCGCACTGCACT	1–2,340
	R: ggctttgttagcagccggatcTCACGACGTCACTTCGACAGA	
*GE000601*	F: gtgccgcgcggcagccatatgACCCGCTCTTCTCTCAACTCC	1–2,526
	R: ggctttgttagcagccggatcTCATGCTTGGATCGATTCTGT	

Lowercase letters refer to the vector sequence and uppercase letters refer to the gene sequence.

Vector constructs were transformed into *E. coli* TOP10 and incubated overnight at 37^°^C on LB agar with ampicillin (100 μg/mL). Recombinant colonies were picked from plates and cultivated in LB broth with appropriate antibiotics at 37^°^C overnight with shaking. Recombinant plasmids were purified using the Plasmid Mini kit I (Omega) and verified by DNA sequencing before the production of recombinant enzymes.

For enzyme production, recombinant plasmids (pET15b-000599/000600/000601) were transformed into competent *E. coli* BL21(DE3)-LysS and incubated overnight at 37^°^C on LB agar with ampicillin (100 μg/mL) and chloramphenicol (30 μg/mL). Single colonies were picked from the plates and cultivated at 37^°^C with shaking in 20 mL LB broth with appropriate antibiotics (Mikkel et al., 2018). Isopropyl-β-D-thiogalactopyranoside (IPTG) with a final concentration of 0.5 mM was added to the culture when the optical density at 600 nm (OD_600_) was 0.3–0.6, and the induction culture was continued at 16^°^C for 12–16 h. Cells were collected by centrifugation and resuspended in 0.5 mL lysis buffer [50 mM *N*-(2-hydroxyethyl) piperazine-*N’*-ethane sulfonic acid (HEPES), pH 7.5, 300 mM NaCl, 2 mM Dithiothreitol (DTT), 1 mM phenylmethylsulfonyl fluoride (PMSF), 1 mM DNAse]. The cells were broken by sonication, and the supernatant was collected after centrifugation at 18,000 rpm for 30 min at 4°C. Subsequently, 3 mL balanced Ni-beads (Sigma-Aldrich) were added slowly at 4^°^C to fully bind the protein sample. Then, 15 mL solution A (50 mM HEPES pH 7.5, 300 mM NaCl) was added for the first wash, followed by 20 mL solution B (50 mM HEPES pH 7.5, 300 mM NaCl, 50 mM imidazole) for the second wash. Finally, 10 mL solution C (50 mM HEPES pH 7.5, 300 mM NaCl, 200 mM imidazole) was added for elution and the eluate was collected. Proteins in the eluate were analyzed by 10% sodium dodecyl sulfate–polyacrylamide gel electrophoresis (SDS-PAGE).

### Enzyme reactions and activity visualization

Recombinant enzymes were screened for activity against 4-nitrophenyl α-L-rhamnopyranoside (*p*NP) glycosides, including *p*NP-α-L-rhamnopyranoside, *p*NP-β-D-galactopyranoside, and *p*NP-β-D-glucopyranoside (Yuanye, China) and against the potato SGAs α-chaconine and α-solanine (Extrasynthese, France). For testing the enzyme activity against 1 mM *p*NP-glycosides, 5 μL of 5 mg/mL recombinant enzyme was added to 95 μL HEPES buffer (pH 7.5) and incubated at 25°C for 1 h, and absorbance at 405 nm was measured using an EPOCH microplate spectrometer (Hennessy et al., 2020). One unit (U) of enzyme activity was defined as the 0.01 change in absorbance value at 405 nm per minute per mg of recombinant enzyme in 100 μL of 50 mM HEPES buffer (pH 7.5) containing 1 mM *p*NP glycosides. For every sample, the activity was measured three separate times (Li et al., 2019).

For testing enzyme activity against SGAs substrates, the recombinant enzyme sample was diluted to 5 mg/mL with 50 mM HEPES. For α-solanine, 5 μL each recombinant enzyme (RhaA alone, GluA alone, GalA alone, RhaA + GluA, RhaA + GalA, GluA + GalA, and RhaA + GluA + GalA) was added to 95, 90, or 85 μL HEPES buffer (pH 7.5) containing 100 μg/mL α-solanine to give a total reaction volume of 100 μL, then reactions were incubated at 25°C for 1 h. A control reaction was included and comprised 100 μL of 50 mM HEPES buffer containing 100 μg/mL α-solanine. Thus, there were a total of eight treatments, each treatment had three replicates. For α-chaconine, 5 μL recombinant enzyme (RhaA alone, GluA alone, RhaA + GluA) was added to 95 μL or 90 μL HEPES buffer (pH 7.5) containing 100 μg/mL α-chaconine and was incubated at 25°C for 1 h. The control reaction comprised 100 μL of 50 mM HEPES buffer containing 100 μg/mL α-chaconine. Thus, there were a total of four treatments and each treatment had three replicates.

Samples were analyzed on a liquid chromatography mass spectrometry (LCMS)-8040 system (Shimadzu) equipped with a Shim-pack XR-ODS III column. At a flow rate of 0.3 mL/min, 10 μL of each sample was injected onto an ODS column (2.0 mm ID × 75 mm length, 1.6 μm diameter; Shim-pack). The mobile phase was composed of solvent A (0.05% formic acid in water) and solvent B (acetonitrile) used in a gradient mode for the separation. A negative electrospray ionization mode was used for detection (α-solanine m/z 868.05, β_1_-solanine m/z 706.4, β_2_-solanine m/z 722.4, γ-solanine m/z 560.4, α-chaconine m/z 852.10, β-chaconine m/z 706.4, γ-chaconine m/z 560.4, solanidine m/z 396.90) (Jensen et al., 2009; Wang et al., 2022). An ion with a specific mass-to-charge ratio generated from the internal standard (the parent ion) was selected and fragmented to obtain its daughter ions. A specific daughter ion was used for generating the chromatogram of the corresponding compound. The peak areas of internal standards and those of the respective target compounds were obtained. The concentration of each compound was quantified by comparing its peak area with the peak area of its respective internal standard (Wang et al., 2022). Levels of α-solanine and α-chaconine were determined by a standard curve.

### Statistical analysis

All analyses were performed using Data Processing System (DPS) software version 14.0 (Tang and Feng, 2012). Differences between two groups were compared using Student’s *t*-test, with *P* < 0.05 considered a significant difference. Differences between more than two groups were analyzed by one-way analysis of repeated measures with Duncan’s multiple comparisons, with *P* < 0.05 considered a significant difference. The statistical data were plotted using GraphPad Prism 8.0 (GraphPad Software, Inc., San Diego, CA, USA). Data are presented as the means ± standard deviation (SD) of three independent experiments.

## Results

### Three glycosidic hydrolase genes were cloned and the recombinant enzyme was purified

**FIGURE 1:**
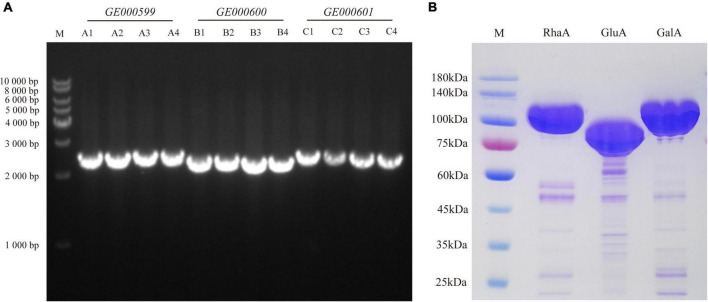
Cloning of glycoside hydrolase genes (*GE000599*, *GE000600*, and *GE000601*) **(A)** and expression of RhaA, GluA, and GalA in *E. coli* BL21 (DE3)-LysS **(B)**.

To examine the function of the enzyme-encoding genes, three genes—*GE000599*, *GE000600*, and *GE000601*—were cloned into *E. coli* from the genome of *G. halophytocola* S2. The lengths of these genes were 2,487, 2,340, and 2,526 bp, respectively (Figure 1A). The purified recombinant proteins RhaA, GluA, and GalA, respectively, were analyzed by 10% SDS-PAGE, and the sizes were approximately 93, 87, and 95 kDa, respectively (Figure 1B).

### The three enzymes showed a variety of substrate hydrolytic activities

**FIGURE 2:**
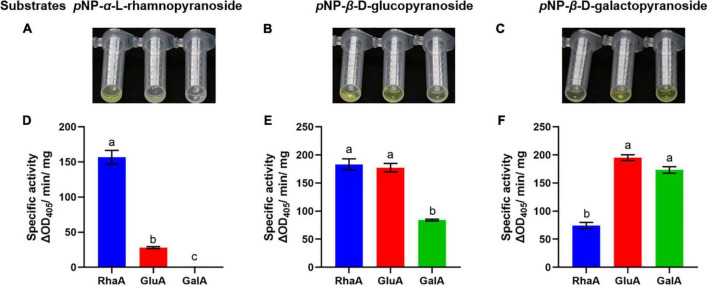
Color reaction and specific activity of recombinant enzymes on the substrates *p*NP-α-L-rhamnopyranoside **(A,D)**, *p*NP-β-D-glucopyranoside **(B,E)** and *p*NP-β-D-galactopyranoside **(C,F)**. Bars (mean ± SD) labeled with different letters within each treatment are significantly different (*P* < 0.05, Duncan’s multiple comparison, *n* = 3).

The purified recombinant enzymes expressed from the three GA-degrading genes were tested with various *p*NP-glycosides, including substrates of α-rhamnosidase, β-glucosidase, and β-galactosidase, to confirm the activities of the three enzymes (Figure 2). Both RhaA and GluA showed hydrolytic activity on *p*NP-α-L-rhamnopyranoside, *p*NP-β-D-glucopyranoside, and *p*NP-β-D-galactopyranoside. RhaA exhibited high hydrolytic activity against *p*NP-α-L-rhamnopyranoside and *p*NP-β-D-glucopyranoside, but weak hydrolytic activity against *p*NP-β-D-galactopyranoside, while GluA exhibited high hydrolytic activity on *p*NP-β-D-glucopyranoside and *p*NP-β-D-galactopyranoside, but weak hydrolytic activity on *p*NP-α-L-rhamnopyranoside. GalA exhibited hydrolytic activity against both *p*NP-β-D-glucopyranoside and *p*NP-β-D-galactopyranoside, but the hydrolysis activity on *p*NP-β-D-galactopyranoside was stronger compared with that on *p*NP-β-D-glucopyranoside. These results indicated that the purified enzymes had high hydrolytic activity without obvious specificity.

### The three enzymes could degrade α-chaconine and α-solanine alone or jointly

**FIGURE 3:**
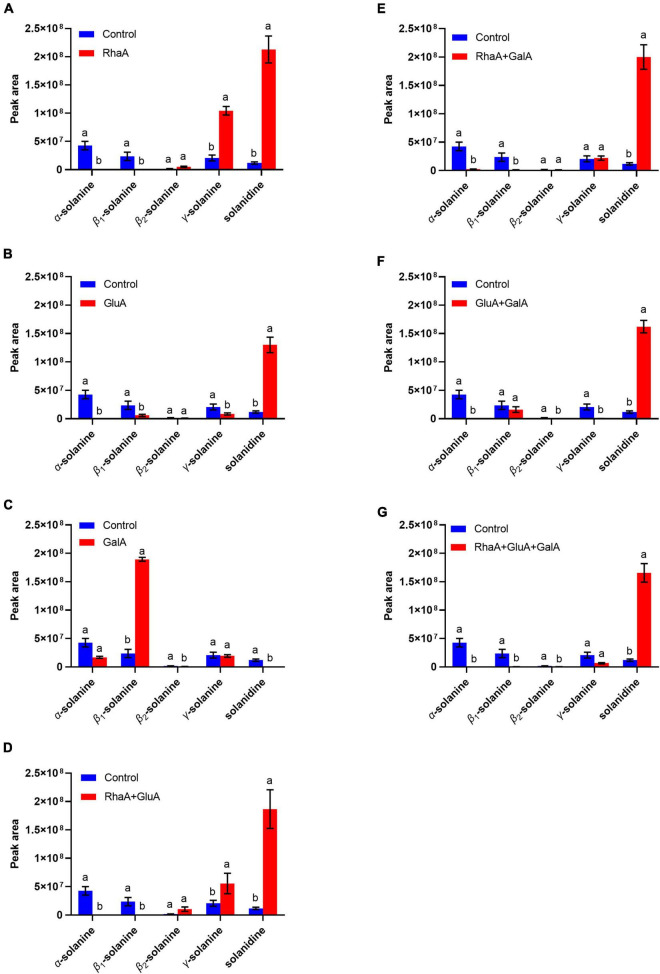
Hydrolysis products released from α-solanine standards by the action of recombinant enzymes of **(A)** RhaA; **(B)** GluA; **(C)** GalA; **(D)** RhaA + GluA; **(E)** RhaA + GalA; **(F)** GluA + GalA; **(G)** RhaA + GluA + GalA. Bars (mean ± SD) labeled with different letters within each treatment are significantly different (*P* < 0.05, independent-sample *t*-tests, *n* = 3).

**FIGURE 4:**
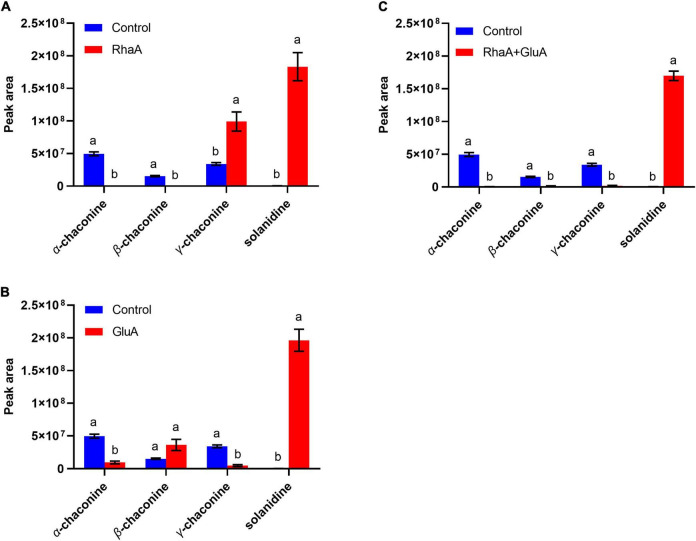
Hydrolysis products released from α-chaconine standards by the action of recombinant enzymes of **(A)** RhaA; **(B)** GluA; **(C)** RhaA + GluA. Bars (mean ± SD) labeled with different letters within each treatment are significantly different (*P* < 0.05, independent-sample *t*-tests, *n* = 3).

Based on the LC-MS analysis, three monosaccharides from both α-chaconine and α-solanine appear to be removed, producing the intermediate compounds β-chaconine, γ-chaconine, β_1_-solanine, and γ-solanine, respectively (Figures 3, 4). Compared with the control, the peak areas of α-solanine, β_1_-solanine, α-chaconine, and β-chaconine were significantly reduced after addition of RhaA (α-solanine: *F* = 62.40, *P* < 0.05; β_1_-solanine: *F* = 20.47, *P* < 0.05; α-chaconine: *F* = 547.09, *P* < 0.05; β-chaconine: *F* = 577.05, *P* < 0.05), while the peak areas of γ-solanine, γ-chaconine, and solanidine were significantly increased (γ-solanine: *F* = 104.79, *P* < 0.05; α-solanine—solanidine: *F* = 128.04, *P* < 0.05; γ-chaconine: *F* = 38.70, *P* < 0.05; α-chaconine—solanidine: *F* = 143.95, *P* < 0.05) (Figures 3A, 4A). The reason for this is that RhaA has high hydrolytic activity on α-L-rhamnopyranoside and β-D-glucopyranoside, but low hydrolytic activity on β-D-galactopyranoside, resulting in partial accumulation of γ-solanine. However, the solanidine production results demonstrate that adding RhaA alone can remove three oligosaccharides from α-chaconine and α-solanine.

Following the addition of GluA, the peak areas of α-solanine, β_1_-solanine, γ-solanine, α-chaconine, and γ-chaconine were significantly reduced (α-solanine: *F* = 63.47, *P* < 0.05; β_1_-solanine: *F* = 21.8, *P* < 0.05; γ-solanine: *F* = 24.52, *P* < 0.05; α-chaconine: *F* = 289.06, *P* < 0.05; γ-chaconine: *F* = 167.84, *P* < 0.05), while that of solanidine was significantly increased (*F* = 272.46; *P* < 0.05) (Figures 3B, 4B). These results indicate that the addition of GluA alone can also remove three oligosaccharides from α-chaconine and α-solanine. The addition of GalA alone produced a large amount of the intermediate β_1_-solanine (*F* = 1304.30, *P* < 0.05) (Figure 3C). GalA has high hydrolysis activity on β-D-glucopyranoside and therefore it cannot hydrolyze α-L-rhamnopyranoside, leading to an accumulation of β_1_-solanine. The peak area of the solanidine was significantly reduced (*F* = 61.35, *P* < 0.05), indicating that GalA might degrade solanidine.

Following the addition of RhaA and GluA, the peak areas of α-solanine, β_1_-solanine, α-chaconine, β-chaconine, and γ-chaconine decreased significantly (α-solanine: *F* = 63.50, *P* < 0.05; β_1_-solanine: *F* = 19.62, *P* < 0.05; α-chaconine: *F* = 553.784, *P* < 0.05; β-chaconine: *F* = 1310.155, *P* < 0.05; γ-chaconine: *F* = 555.149, *P* < 0.05), and the peak areas of γ-solanine and solanidine significantly increased (γ-solanine: *F* = 36.90, *P* < 0.05; α-solanine—solanidine: *F* = 56.132, *P* < 0.05; α-chaconine—solanidine: *F* = 31.443, *P* < 0.05) (Figures 3D, 4C). This is because RhaA has high hydrolytic activity on α-L-rhamnopyranoside and β-D-glucopyranoside, but low hydrolytic activity on β-D-galactopyranoside, resulting in partial accumulation of γ-solanine.

With the addition of RhaA and GalA, the peak areas of α-solanine and β_1_-solanine decreased significantly (α-solanine: *F* = 55.30, *P* < 0.05; β_1_-solanine: *F* = 20.52, *P* < 0.05), while that of solanidine was significantly increased (*F* = 152.01, *P* < 0.05) (Figure 3E). And after the addition of GluA and GalA, the peak areas of α-solanine and γ-solanine decreased significantly (α-solanine: *F* = 63.40, *P* < 0.05; γ-solanine: *F* = 28.62, *P* < 0.05), while that of solanidine was significantly increased (*F* = 287.40, *P* < 0.05) (Figure 3F). When all three enzymes (RhaA, GluA, and GalA) were added together, the peak areas of α-solanine, β_1_-solanine, and γ-solanine all decreased (α-solanine: *F* = 63.664, *P* < 0.05; β_1_-solanine: *F* = 21.121, *P* < 0.05; γ-solanine: *F* = 54.952, *P* < 0.05) and the peak areas of the final product solanidine increased significantly (*F* = 219.54, *P* < 0.05) (Figure 3G).

## Discussion

Enzyme mining is a promising way to compensate for the insufficiency of present catalysts (Nestl et al., 2011), and exploiting microorganism resources is an important strategy in this approach (Aharoni, 2009; Singh et al., 2019; Cheng et al., 2022). α-L-Rhamnosidase, β-D-galactosidase, and β-D-glucosidase are three common glycosidic hydrolases, which belong to the GH78, GH2, and GH3 glycosidic hydrolase families, respectively (Dong et al., 2021; Cheng et al., 2022). These three enzymes can be obtained from various sources such as microorganisms, plants, and animals. However, according to their source, their properties of the enzymes differ markedly depending on their source (Finocchiaro et al., 1980; Cheng et al., 2021). Compared with other available sources, microorganisms offer numerous advantages including easy handling, higher multiplication rate, and high production yield. Recently, these enzymes have been isolated and purified from a wide range of microorganisms (Panesar et al., 2010; Emanuelle et al., 2022; Kim et al., 2022). In our previous study, we first identified a gene cluster encoding α-L-rhamnosidase, β-D-galactosidase, and β-D-glucosidase in *G. halophytocola* S2 (Wang et al., 2022). In the current study, three genes (*GE000599*, *GE000600*, and *GE000601*) in the identified gene cluster were cloned and recombinant proteins were expressed on the basis of previous studies, and then the multifunctional activity of these three enzymes was confirmed.

**FIGURE 5:**
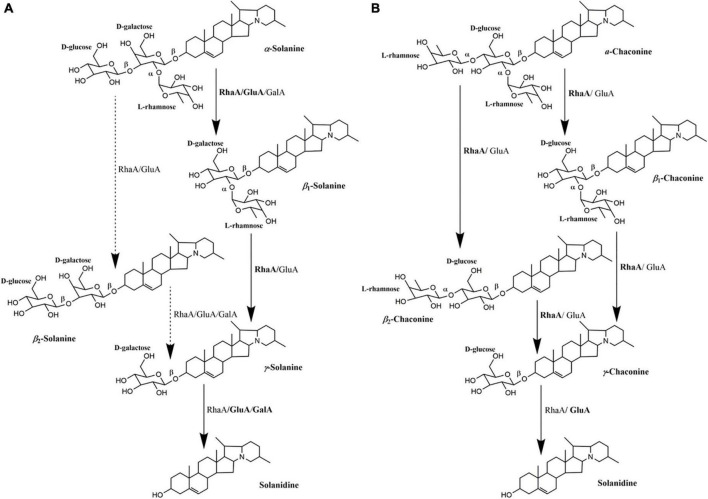
Multifunctional roles of glycoside hydrolases (RhaA, GluA, and GalA) produced by *Glutamicibacter halophytocola* S2 in the degradation of α-solanine **(A)** and α-chaconine **(B)**. The degradation process of α-solanine and α-chaconine referred to Jensen et al. (2009). Solid arrows represent the proposed main pathway of *G. halophytocola* S2. The dashed arrow represents the secondary catabolic pathway. Enzyme names to the right of each arrow is the enzyme that can act on that site.

On the basis of the degradation process of α-solanine and α-chaconine summarized by Jensen et al. (2009), the main pathways of degradation of α-solanine by *G. halophytocola* S2 could be summarized (Figure 5A, right). The first step of degradation of α-solanine by *G. halophytocola* S2 is to remove the β-glucose on the side chain and generate β_1_-solanine. In this step, RhaA, GluA, and GalA can all participate, but RhaA and GluA are the key enzymes. In the second step, α-rhamnose was removed to generate γ-solanine. Both RhaA and GluA can participate in this step, but RhaA is the key enzyme. The final step is the removal of β-galactose to produce solanidine. RhaA, GluA, and GalA can all participate in this step, but GluA and GalA are key enzymes. The small amount of β_2_-solanine generated in some of the treatments in the present study (Figures 3A,D) indicates that there may be a secondary degradation pathway for the breakdown of α-solanine by *G. halophytocola* S2 (Figure 5A, left). This secondary pathway involves the removal of α-rhamnose from the side chain of α-solanine as the first step, followed by the removal of β-glucose to generate γ-solanine in the second step. In addition, the pathway of degradation of α-chaconine by *G. halophytocola* S2 can also be summarized and is consistent with many reports (Figure 5B; Friedman et al., 1993; Oda et al., 2002; Koffi et al., 2017). However, the present study found that RhaA and GluA alone could also degrade α-chaconine, that is, the first step was to remove any rhamnose on the side chain of α-chaconine to produce β_1_-chaconine, and the second step was to remove another rhamnose to produce β_2_-chaconine. Both RhaA and GluA can participate in these two steps, and RhaA is the key enzyme. Since the m/z of β_1_-chaconine and β_2_-chaconine are the same, these compounds were uniformly called β-chaconine in LC-MS analysis. In the final step of the pathway, RhaA and GluA can also participate in the removal of β-glucose, but GluA is the key enzyme.

The crude protein extract produced by potato pathogenic fungus *Gibberella pulicaris* could degrade α-chaconine and α-solanine, but it was strain specific. For example, strain R-7843 could only degrade α-chaconine, while strain R-6380 could only degrade α-solanine (Weltring et al., 1997). Hennessy et al. (2020) found that the enzyme activities of the three enzymes encoding by the gene cluster in *Arthrobacter* sp. S41 were involved in the complete deglycosylation of α-chaconine and α-solanine, but the specific functions and degradation pathways of each enzyme were unclear. The present study characterized the multifunctional activities and specific degradation pathways of these three enzymes in *G. halophytocola* S2. This clarified the molecular mechanism of degradation of α-chaconine and α-solanine by *G. halophytocola* S2 in the gut of *P. operculella*. Furthermore, in this study, RhaA, and GluA alone were found to degrade α-solanine and α-chaconine, which had not been reported. Therefore, *G. halophytocola* has potential to be used to remove α-chaconine and α-solanine in potato foods and is advantageous owing to the high degradation activity, green environmental protection, and a very broad application prospect. The multifunctional enzyme produced by this strain have high glycosidic hydrolase activity and clear sequence information, providing an effective and eco-friendly method for solanidine production. It can reduce the consumption of acid/base, hazardous organic reagents, and raw materials, and has great development potential. Further work will be conducted on product research on the two recombinant enzymes RhaA and GluA. In addition, the next step will be to predict the potential catalytic residues of the enzymes and the binding mechanism of substrates through computational analysis, and aim to understand the catalytic effect of the enzymes on substrates at the molecular level. This will lay a theoretical foundation for the improvement of enzyme properties through protein engineering in the future.

## Conclusion

The three enzymes produced by *G. halophytocola* S2 are multifunctional and result in the efficient degradation of α-solanine and α-chaconine by *G. halophytocola* S2. This study not only clarified the molecular mechanism of *G. halophytocola* S2 degradation of α-solanine and α-chaconine but also identified the specific steps in the degradation of α-solanine and α-chaconine by three deglycosylation enzymes. These findings contribute to understanding of the detoxification mechanism of insect gut microbes. Simultaneously, the three multifunctional enzymes have high glycosidic hydrolysis activity and clear gene sequence information, and thus also have application potential in removing toxic SGAs from potato and other edible plants of the family Solanaceae.

## Data availability statement

The datasets presented in this study can be found in online repositories. The names of the repository/repositories and accession number(s) can be found below: https://www.ncbi.nlm.nih.gov/genbank/, CP102487.

## Author contributions

GX and BC conceived the idea, edited the manuscript, and made substantial contribution to the conception of the work. WW and GD performed gene cloning and protein expression and purification, respectively. WW, GY, and KZ performed enzyme activity measurement and SGAs degradation activity measurement. WW conducted statistical analyses and wrote the original manuscript. All authors have read and approved the content of the manuscript and contributed to the article and approved the submitted version.
